# Engaging with young professionals in transfusion medicine: Insights from a needs assessment survey targeting an international cohort

**DOI:** 10.1111/vox.13783

**Published:** 2025-01-12

**Authors:** Eunike C. McGowan, Antoine Lewin, Jian Ou‐Yang, Saikat Mandal, Nour Almozain, Abiy Belay Ambaye, Jannison Karlly Cavalcante Ribeiro, Lilian Antwi Boateng, Carla Luana Dinardo, Praiseldy Langi Sasongko, the Young Professionals Council

**Affiliations:** ^1^ Division of Hematology and Transfusion Medicine, Department of Laboratory Medicine Lund University Lund Sweden; ^2^ Medical Affairs and Innovation, Héma‐Québec Montreal Quebec Canada; ^3^ Guangzhou Blood Center Guangzhou China; ^4^ Medical Oncology, Hull York Medical School University of Hull Hull UK; ^5^ Department of Pathology and Laboratory Medicine King Faisal Specialist Hospital and Research Centre Riyadh Saudi Arabia; ^6^ Department of Pathology, College of Medicine King Saud University Riyadh Saudi Arabia; ^7^ Ethiopian Blood and Tissue Bank Service Addis Ababda Ethiopia; ^8^ Centro de Hematologia e Hemoterapia do Ceará – Hemoce Fortaleza Brazil; ^9^ Instituto Pró‐Hemo Saúde – IPH Fortaleza Brazil; ^10^ Department of Medical Diagnostics Kwame Nkrumah University of Science and Technology Kumasi Ghana; ^11^ Immunohaematology Laboratory University Health Services, Kwame Nkrumah University of Science and Technology Kumasi Ghana; ^12^ Immunohaematology Division Fundação Pró‐Sangue São Paulo Brazil; ^13^ Department of Public and Occupational Health Amsterdam UMC, location VUmc Amsterdam The Netherlands

**Keywords:** education, engagement, transfusion medicine, young professionals

## Abstract

**Background and Objectives:**

Professionals who work or study in transfusion medicine under 40 years of age are considered young professionals (YPs) by the International Society of Blood Transfusion (ISBT). While the ISBT provides opportunities for YPs, their needs have to be assessed to customize initiatives in a way that could potentially improve their engagement. This survey aimed to assess the needs of YPs in transfusion medicine and understand their perspectives on future ISBT initiatives.

**Materials and Methods:**

Between January and February 2023, a 28‐question online survey was accessible through a generalized link across the ISBT network. Skip‐logic responses from 352 YPs, including 151 ISBT members and 201 non‐members, were analysed. Each question varied in the number of responders and, consequently, the number of responses.

**Results:**

Firstly, the most important needs of YPs from the survey were educational opportunities and training programmes, with 70% of respondents indicating for educational content in specific fields of transfusion. Secondly, *Transfusion Today* published by the ISBT (46.9%) ranked the highest in engagement, while ISBT Academy and Academy funding ranked the lowest (12.8%). ISBT members reported attending ISBT activities or using ISBT resources more often than non‐members, although this was not statistically significant. The primary barrier preventing both non‐members and ISBT members from engaging in ISBT activities was a lack of awareness.

**Conclusion:**

Raising awareness on a regular basis, a customized communication style (e.g., by a representative or different languages) and activities for non‐members may be key to improving YP engagement and expanding the ISBT network.

**Highlights**

- This survey aimed to assess the needs of young professionals globally, which included International Society of Blood Transfusion (ISBT) members and non‐members from all World Health Organization regions.
- Findings showed that the primary reason for a lack of engagement in ISBT activities was a lack of awareness from both members and non‐members.
- Engagement can be improved by raising awareness of ISBT activities, along with customizing transfusion‐related educational content.

## INTRODUCTION

The Young Professionals Council (YPC) of the International Society of Blood Transfusion (ISBT) was founded in 2018 to advocate for the needs of young professionals (YPs) in transfusion medicine. YPC includes elected representatives from all seven World Health Organization (WHO) regions. In an election, YPs who are working or studying in transfusion medicine and are 40 years old or less are encouraged to apply and are then selected by the YPC members through a score‐based criteria and, in some cases, an interview [1]. YPC aims to foster ambition and build a community among YPs starting a career in transfusion medicine [1]. Since its inception, YPC has launched many initiatives to fulfil this mission, including social media campaigns, face‐to‐face events, a survey to capture YP experiences during COVID‐19 and the development of new opportunities for involvement within the ISBT [1, 2, 3].

Given the growing YP community, it is necessary to comprehend the needs of YPs to ensure that future activities will nurture their potential. Therefore, this survey aimed to assess the needs of YPs in transfusion medicine and to understand their perspectives on future YPC and ISBT initiatives.

## MATERIALS AND METHODS

Between January and February 2023, a 28‐question online survey in English language was accessible through a generalized link across the ISBT network (Table S1). Potential participants were made aware of the survey by (1) a section in the ‘Opportunities for Young Professionals’ of the YPC page on the ISBT website; (2) YPC representatives promoting the link to their respective region either directly or through social platforms (e.g., WhatsApp groups) and/or a request via email through the networks of other ISBT members (e.g., ISBT regional directors) and (3) two social media posts in January 2023 through the ISBT account. The first question was an age check that ensured only participants aged 40 or under (‘young professionals’) completed the survey—whether they be ISBT members or non‐members.

The rest of the survey consisted of questions on membership involvement and satisfaction, and was divided in five categories: communications clarity, engaging with the community, education, congresses and future involvement. These took the form of multiple‐choice questions, open‐ended questions and free text fields (to collect a diversity of opinions). The survey had a skip‐logic method dependent upon respondents' previous response(s), and no questions were mandatory (except the first one on age).

The results were analysed descriptively. The analysed responses included only those from participants who consented to participate and have their answers disseminated (anonymously) for research purposes. *Z*‐tests for two proportions were used to compare responses between members and non‐members. Significance level was set to <0.01 to account for test multiplicity and to control the false discovery rate.

## RESULTS

### Participant characteristics

Overall, 95.4% (*n* = 352) YPs consented for the publication of their responses (in addition to first one on age), including 151 ISBT members and 201 non‐members. The most predominant WHO region in the sample were Southeast Asia (147/316 [46.5%]) and the Western Pacific region (54/316 [17.1%]; Table 1). Among those who responded, the three most common primarily roles were physician (86 [27.2%]), allied health care professionals (66 [20.9%]) and graduate students or postgraduate professionals (64 [20.3%]). Most participants (*N* = 187 [59.2%]) had five or more years of experience in transfusion medicine (*N* = 315).

**TABLE 1:** Participant characteristics.

	ISBT members	Non‐members	Total
	*N* = 151	*N* = 165	*N* = 316
WHO region			
Southeast Asia	74 (49.0%)	73 (44.2%)	147 (46.5%)
Western Pacific	18 (11.9%)	36 (21.8%)	54 (17.1%)
Europe	19 (12.6%)	8 (4.8%)	27 (8.5%)
Africa	9 (6%)	16 (9.7%)	25 (7.9%)
Central and South America	12 (7.9%)	12 (7.3%)	24 (7.6%)
Eastern Mediterranean	11 (7.3%)	10 (6.1%)	21 (6.6%)
North America	8 (5.3%)	10 (6.1%)	18 (5.7%)

	*N* = 151	*N* = 165	*N* = 316
Primary role			
Physician	53 (35.1%)	33 (20%)	86 (27.2%)
Allied healthcare professional^a^	21 (13.9%)	45 (27.3%)	66 (20.9%)
Graduate or postgraduate student	33 (21.9%)	31 (18.8%)	64 (20.3%)
Scientist	27 (17.9%)	34 (20.6%)	61 (19.3%)
Undergraduate student	1 (0.7%)	4 (2.4%)	5 (1.6%)
Nurse	1 (0.7%)	2 (1.2%)	3 (0.9%)
Other	15 (9.9%)	16 (9.7%)	31 (9.8%)

	*N* = 151	*N* = 164	*N* = 315
Years of experience in transfusion medicine			
1–2	23 (15.2%)	46 (28.0%)	69 (21.9%)
3–4	34 (22.5%)	25 (15.2%)	59 (18.7%)
≥5	94 (62.3%)	93 (56.7%)	187 (59.4%)

Abbreviations: ISBT, International Society of Blood Transfusion; WHO, World Health Organization.

^a^ Included one medical laboratory technician who had attended a diploma or certificate course in medical laboratory technology.

### Engagement in current scientific activities

A larger proportion of participants reported attending the following activities at least occasionally: reading *Transfusion Today* (105/224 [46.9%]) and reading or submitting an article to *Vox Sanguinis* (94/211 [44.5%]) and ISBT Education (76/230 [33.0%]; Table S2). In contrast, there were fewer participants who reported the use of the following activities at least occasionally: ISBT congresses (60/218 [27.5%]), the ‘I TRY IT’ programme for YPs with little or no research experience to further learn and develop research skills [4] (50/234 [21.4%]), activities for YPs at ISBT congresses (41/238 [17.2%]) and ISBT Academy which consists of the e‐learning platform ISBT Education, educational projects and reviews of applications for educational or research activities for Academy funding [5, 6] (31/242 [12.8%]). Although non‐significant, there were differences from participants who indicated ISBT membership and non‐members, with more ISBT members than non‐members reported feeling part of the ‘global ISBT/YP community’, while non‐members showed a higher percentage of rarely or never participating in or accessing these activities and resources (Tables 2 and S2).

**TABLE 2:** Participant responses to questions about their engagement as young professionals.

Questions	Total	ISBT members	Non‐members	*p*‐value^d^
	*N* = 249	*N* = 128	*N* = 121	
Do you feel like you are part of the global ISBT YPs community?				
Yes	75 (30.1%)	45 (35.2%)	30 (24.8%)	0.0750
No	52 (20.9%)	25 (19.5%)	27 (22.3%)	0.5892
Not sure	122 (49.0%)	58 (45.3%)	64 (52.9%)	0.2301

	*N* = 51, 81 responses	*N* = 24, 35 responses	*N* = 27, 46 responses	
If ‘no’, what factors prevent you from feeling as such?				
Cost of ISBT membership	26 (51.0%)	9 (37.5%)	17 (63.0%)	0.0688
Lack of time to attend events	25 (49.0%)	11 (45.8%)	14 (51.9%)	0.6672
Language or cultural barriers	8 (15.7%)	1 (4.2%)	7 (25.9%)	0.0331
Other^a^	22 (43.1%)	14 (58.3%)	8 (29.6%)	0.0385

	*N* = 196	*N* = 97	*N* = 99	
Why didn't they engage with congress/activities/resources?^b^				
Not aware	89 (45.4%)	38 (39.2%)	51 (51.5%)	0.0836
Non‐member/new member^c^	25 (12.8%)	7 (7.2%)	18 (18.2%)	0.0214
No time	40 (20.4%)	30 (30.9%)	10 (10.1%)	0.0003
Not accessible	11 (5.6%)	3 (3.1%)	8 (8.1%)	0.1285
Financial reasons	17 (8.7%)	10 (10.3%)	7 (7.1%)	0.4178
Not relevant	12 (6.1%)	9 (9.3%)	3 (3.0%)	0.0697
Language barriers	2 (1.0%)	0	2 (2.0%)	0.1585

	*N* = 236, 633 responses	*N* = 123, 330 responses	*N* = 113, 303 responses	
How can we better engage and connect with you?				
Provide regular updates	166 (70.3%)	93 (75.6%)	73 (64.6%)	0.0643
Allow for non‐members to join different activities, like webinars	148 (62.7%)	59 (47.9%)	89 (78.8%)	<0.0001
Get to know your YP regional representative; better connect with him/her	126 (53.4%)	80 (65.0%)	46 (40.7%)	0.0002
Improve content on social media	108 (45.8%)	57 (46.3%)	51 (45.1%)	0.1862
Provide content in different languages	73 (30.9%)	32 (26.0%)	41 (36.3%)	0.0891
Other	12 (5.1%)	9 (7.3%)	3 (2.7%)	0.1031

	*N* = 153	*N* = 80	*N* = 73	
What educational content would you like or need more of?^b^				
Transfusion‐field relevant content, courses and basics	104 (68.0%)	56 (70.0%)	48 (65.8%)	0.5755
Guidelines, protocols or latest advances in various fields of transfusion, including technological advances	20 (13.1%)	11 (13.8%)	9 (12.3%)	0.7949
Platform(s) to share experiences or have discussions about ideas of the field from different regions (e.g., in webinars or podcasts)	18 (11.8%)	9 (11.3%)	9 (12.3%)	0.8337
Career growth and skill development	8 (5.2%)	2 (2.5%)	6 (8.2%)	0.1118
Not sure	3 (2.0%)	2 (2.5%)	1 (1.4%)	0.6171

Abbreviations: ISBT, International Society of Blood Transfusion; YP, young professional.

^a^ Responses included time zone differences, opportunities and lack of event diversity, relevance and awareness.

^b^ Open‐ended question and answers were categorized accordingly.

^c^ In the free‐text option, new members described themselves as ‘new members’ of the ISBT, new to the website, and/or very early in their career. Both new members and non‐members stated their (lack of) membership as the reason why they do not engage.

^d^ *Z*‐tests for two proportions were used. The significance level was set to <0.01 to account for test multiplicity and to control the false discovery rate.

Follow‐up open‐question responses showed that the most common barrier for engagement was a lack of awareness for non‐members (51.5%) and ISBT members (39.2%; Table 2). For ISBT members, this was followed by a lack of time (30.9%) and financial reasons (10.3%). For non‐members, this was because of no membership (18.2%) and the lack of time (10.1%; Table 2). Differences between member and non‐member were statistically significant only for the lack of time parameter (30.9% vs. 10.1%, respectively, *p* = 0.0003).

Respondents showed interest for engaging with the YPC/ISBT community for the following activities: attending educational webinars (198/236 [83.9%]), attending certificate programmes (170/236 [72.0%]), attending networking activities (159/236 [67.4%]) and learning about new opportunities and resources for research collaborations (155/236 [65.7%]; Table S3). No statistical differences were observed between members and non‐members.

There were 4 non‐members and 34 ISBT members who indicated membership in a Working Party, comprising 16% of the total respondents (importantly, formal participation in a Working Party requires ISBT membership). The majority of this group (29/38, 76.3%) indicated satisfaction with their Working Party membership, while those who were not satisfied (*N* = 9/38) indicated in a multiple‐answer question that engaging with experts (66.7%), contributing to Wikipedia or similar (44.4%), designing webinars/live journal clubs (33.3%) and voicing thoughts/ideas (22.2%) would improve their experience.

### Barriers and facilitators to further engaging with the ISBT community

Overall, 20.9% (52/249) of participants reported *not* feeling part of the global ISBT/YP community, citing ISBT membership costs (26/51 [51.0%]) and the lack of time (25/51 [49.0%]) as key barriers to feeling as such (Table 2), while 49% (122/249) stated they were ‘not sure’ what it means to be part of such a community. Most reported needing more educational content, courses and basics related to a specific field within transfusion (104/153 [68.0%]). Less than 15% reported needing more educational content on career growth and skill development (8/153 [5.2%]), online platforms to share experiences (18/153 [11.8%]), or guidelines, protocols and reports of recent advances (20/153 [13.1%]). The participating YPs suggested that the YPC better engage with them by providing regular updates (166/236 [70.3%]), allowing non‐members to join different activities (148/236 [62.7%]), facilitating contacts with their local YPC representative (126/236 [53.4%]) and improving the content disseminated on social media (108/236 [45.8%]). No statistical differences were observed between members and non‐members.

## DISCUSSION

This survey described the extent to which YPs engage with the ISBT/YP community, as well as the barriers and facilitators to further engagement. This investigation was considered important because the previous global survey for YPs in transfusion medicine was related to the psychological impact of COVID‐19 on YPs in transfusion medicine, including an assessment of changes in work routines and degree of organizational support [2]. Overall, at least half of the respondents reported participating at least occasionally in current activities intended for YPs and at least half reported using the resources intended for them at least occasionally. Interestingly, approximately 20% did *not* feel like they were part of the global ISBT/YP community compared to 49% of participants (45% of members and 53% of non‐members) who were ‘not sure’. This could suggest that further actions are needed to formalize the community by providing and inviting YPs to participate more in ISBT YP events and be connected to one another.

With >70% reporting the need for specific educational content, developmental and training needs are applicable not just for the ISBT members but also to the wider transfusion community involved in educational institutions or other professional bodies.

There were a few notable differences between ISBT members and non‐members. To begin, ISBT members reported attending ISBT/YP activities or using YP resources more often than non‐members. Additionally, although non‐significant, more ISBT members than non‐members reported feeling part of the global ISBT/YP community. Not surprisingly, among those who did not feel as such, more non‐members than members cited membership costs as a barrier to further engaging with the ISBT community. Nonetheless, the number of responses from non‐members raises the question on how to bring awareness and involvement in ISBT activities to this group. Currently, specific efforts include social media posts and inclusion in the YP community WhatsApp group where all YP initiatives are regularly posted and open questions and discussions are encouraged.

This survey has some limitations. First, to increase the survey completion rate, eligible participants were free to skip as many questions as they wished to. As a result, no single question was answered by the same set of participants, thus hindering data interpretation. Second, the participation rate could be improved: the 151 ISBT members who consented to the publication of responses represented only 37.8% of the total number ISBT members aged <40 years in mid‐January 2023 (M. Kada, ISBT Central Office, personal communication, 27 May 2024). Lastly, an unexpectedly large proportion of participants were from Southeast Asia, a region that accounts for only 15.5% of ISBT members aged ≤40, suggesting a sampling bias (M. Kada, ISBT Central Office, personal communication, 27 May 2024). It may be beneficial for needs assessment survey to be repeated periodically at various levels (e.g., regionally, nationally and internationally) to further clarify the needs of YPs and monitor those needs as the field of transfusion medicine also evolves.

The results of this survey will help guide the development of future ISBT/YPC initiatives. The preliminary results of this survey were presented to the chairs of the ISBT Working Parties and to the ISBT board members at the International Scientific Advisory Committee meeting in January 2024. Thus far, this survey has catalysed actions including an open‐house meeting for YPs, a YP community WhatsApp group, YP events for upcoming congresses and a concerted push for more YPs to participate in Working Parties [7]. Future initiatives will aim to help address the remaining engagement and educational barriers of YPs to ensure the renewal of expertise in specialized research communities and contribute to future developments in the field of transfusion.

## CONFLICT OF INTEREST STATEMENT

The authors declare no conflicts of interest.

## Supporting information


**Table S1.** Survey questions.
**Table S2**. Activities attended and resources used by participating young professionals.
**Table S3**. Objectives pursued by participants through their engagement with the YPC/ISBT community.

## Data Availability

The data that support the findings of this study are available from the corresponding author upon reasonable request.
