# Supplementary material for: Engaging with young professionals in transfusion medicine: Insights from a needs assessment survey targeting an international cohort
Source: Vox Sang. 2025 Jan 12;120(3):320–5. doi: 10.1111/vox.13783 (PMC11931347; doi:10.1111/vox.13783)
Supplement: Supplementary file 1 — Table S1. Survey questions. Table S2. Activities attended and resources used by participating young professionals. Table S3. Objectives pursued by participants through their engagement with the YPC/ISBT community. [file VOX-120-320-s001.docx]

**Engaging with young professionals in transfusion medicine: Insights from a needs assessment survey targeting an international cohort**

**Supplemental material**

Table S1. Survey questions

| No. | Question | Answer choices |
| --- | --- | --- |
| 1 | Age check: Are you under 40 years old? | - Yes - No |
| 2 | I consent that my data will be used for future publications | - Yes - No |
| 3 | Which WHO region are you from? (Select one) | - North America - Central and South America - Africa - Europe - Eastern Mediterranean - Western Pacific - South East Asia |
| 4 | What is your primary role? (Select one) | - Undergraduate student - Graduate or postgraduate student - Physician - Nurse - Scientist - Allied healthcare professional (medical laboratory technician who has undertaken a diploma or certificate course in medical laboratory technology) - Other (please specify) |
| 5 | Which field(s) in transfusion medicine are you working in? (Multiple answers possible) | - Blood Components - Blood supply management - Donors and donation - Rare donors - Blood products and safety - Clinical transfusion - Transfusion practitioner - Hemovigilance - Transfusion-Transmitted Infection Disease (TTID) - Statistics/Mathematical modelization - Epidemiology - Immunohematology - RBC Immunogenetics and Blood Group Terminology - Platelet immunobiology - Cellular therapies - Quality management - Information Technology - Other (please specify) |
| 6 | For how many years have you been working in Transfusion Medicine? | - 1-2 years - 3-4 years - 5+ years |
| 7 | For how many years have you been a member of ISBT | - 1-2 years - 3-4 years - 5+ years - I am not a member (yet) |
| 8 | Which of the following social media platforms do you actively follow uson? (Multiple answers possible) | - Instagram - Twitter - LinkedIn - Facebook - Weibo - I don’t follow any of these |
| 9 | Since January 2020, how often have you engaged with the followingISBT activities or resources? | - ISBT congresses - YP activities at ISBT congresses - ISBT Academy and Academyfunding - I TRY IT - ISBT Education and/or Educationapp - Reading Transfusion Today - Reading or submitting to Vox Sanguinis |
| 10 | For any of the above that you “never” or “rarely” engaged with, whatis or are the reasons? | Open-text answer |
| 11 | If the following initiatives happened, how often would you use them? | - YP podcasts regarding articles summaries orinterviews with other YPs - Regular email updates from the YPC - Constant updates and links on the YPC website - Using different languages for webinars or thecontent that is published |
| 12 | How can we better customize our communication? | Open-text answer |
| 13 | Do you feel like you are part of the global ISBT Young Professionals(YP) community? | - Yes - No - Not sure what being part of a global ISBT YP community means |
| 14 | If “no,” what factors prevent you from feeling like part of thecommunity? | - Cost of ISBT membership - Lack of time to attend events - Language or cultural barriers - Other (please specify) |
| 15 | How can we better engage and connect with you? (Multiple choices possible) | - Provide content in different languages - Provide regular updates (e.g. email newsletters) - Allow for non-members to join different activities like webinars - Improve content on social media - Get to know your YP regional representative; better/connect with him/her - Other (please specify) |
| 16 | What are you seeking from the Young Professionals Council (YPC)/ISBT community? (Multiple choices possible) | - Networking with other YPs worldwide - Educational webinars for YPs on various topics moderated by an expert - Certificate programs for career growth and skill development - Mentorship - Opportunities and resources for research collaborations - Resources for internship possibilities - Other (please specify |
| 17 | What are important needs of Young Professionals in Transfusion Medicine in your local or regional area? | Open-text answer |
| 18 | Are you a member of an ISBT Working Party? | - Yes - No |
| 19 | If “yes,” Are you satisfied with your involvement in the working party (WP)? | - Yes - No |
| 20 | If “no,” which factors would improve your satisfaction in yourmembership? (Multiple answers possible) | - Becoming a secretary or other position - Design webinars/live journal clubs with the WP and the Young Professionals Council - Contribute towards Wikipedia pages or other places - Being given the time to engage with WP experts - Being able to voice ideas or thoughts during WP meetings - Other (please specify) |
| 21 | Are you interested in becoming a Working Party member? | - Yes - No |
| 22 | If “yes,” which working party are you interested in? Please indicate your top two preferences. | - Blood Components - Blood Supply Management - Cellular Therapies - Clinical Transfusion - Donors and Donation - Global Blood Safety - Granulocyte Immunobiology - Haemovigilance - Immunohaematology - Information Technology - Platelet Immunobiology - Quality Management - Rare donors - Red Cell Immunogenetics and Blood Group Terminology - Transfusion Transmitted Infectious Diseases |
| 23 | What educational content would you like or need more of? | Open-text answer |
| 24 | When attending an ISBT congress, would you be interested in any ofthese activities? | - Young Professionals Breakfast - Young Professionals Drinks - Speed dating with experts - Pitch your research workshop - Scientific writing workshop |
| 25 | Do you have any other ideas for a Young Professional activity at ISBTcongresses? | Open-text answer |
| 26 | For future congresses, what is your preference for the type of congress that you would attend? | - Face-to-face - Virtual - Hybrid - All of the above is fine |
| 27 | We are interested in following up with you through interviews and/or focus groups. Would you like to be involved? | - No - Yes (please provide your email below) |
| 28 | Is there anything else we have missed that’s important for you? Please let us know in the comment box below. | Open-text answer |

**Table S2. Activities attended and resources used by participating young professionals.**

|  | **ISBT  members** | **Non- members** | **Total** |
| --- | --- | --- | --- |
| ISBT congresses | *N=99* | *N=119* | N=218 |
| Rarely or never | 57 (57.6%) | 101 (84.9%) | 158 (72.5%) |
| Sometimes, often or always | 42 (42.4%) | 18 (15.1%) | 60 (27.5%) |
|  |  |  |  |
| YP activities at ISBT congresses | *N=118* | *N=120* | N=238 |
| Rarely or never | 84 (71.2%) | 113 (94.2%) | 197 (82.8%) |
| Sometimes, often or always | 34 (28.8%) | 7 (5.8%) | 41 (17.2%) |
|  |  |  |  |
| ISBT Academy | *N=122* | *N=120* | N=242 |
| Rarely or never | 96 (78.7%) | 115 (95.8%) | 211 (87.2%) |
| Sometimes, often or always | 26 (21.3%) | 5 (4.2%) | 31 (12.8%) |
|  |  |  |  |
| I TRY IT | *N=114* | *N=120* | N=234 |
| Rarely or never | 79 (69.3%) | 105 (87.5%) | 184 (78.6%) |
| Sometimes, often or always | 35 (30.7%) | 15 (12.5%) | 50 (21.4%) |
|  |  |  |  |
| ISBT Education and/or Education app | *N=111* | *N=119* | N=230 |
| Rarely or never | 52 (46.8%) | 102 (85.7%) | 154 (67.0%) |
| Sometimes, often or always | 59 (53.2%) | 17 (14.3%) | 76 (33.0%) |
|  |  |  |  |
| Reading Transfusion Today | *N=111* | *N=113* | N=224 |
| Rarely or never | 40 (36.0%) | 79 (69.9%) | 119 (53.1%) |
| Sometimes, often or always | 71 (64.0%) | 34 (30.1%) | 105 (46.9%) |
|  |  |  |  |
| Reading or submitting to Vox Sanguinis | *N=100* | *N=111* | N=211 |
| Rarely or never | 42 (42.0%) | 75 (67.6%) | 117 (55.5%) |
| Sometimes, often or always | 58 (58.0%) | 36 (32.4%) | 94 (44.5%) |

**Abbreviations:** ISBT=International Society of Blood Transfusion; YP=young professional

**Table S3. Objectives pursued by participants through their engagement with the YPC/ISBT community**

|  | **ISBT members** | **Non-members** | **Total** |
| --- | --- | --- | --- |
| *What are you seeking from the YPC/ISBT community?*  *(Multiple choices possible)* | *N=122*  *546*  *responses* | *N=114*  *384*  *responses* | *N=236*  *930 responses* |
| Educational webinars for YPs on various topics moderated by an expert | 103 (18.9%) | 95 (24.7%) | 198 (21.3%) |
| Certificate programs for career growth and skill development | 96 (17.6%) | 74 (19.3%) | 170 (18.3%) |
| Networking with other YPs worldwide | 98 (17.9%) | 61 (15.9%) | 159 (17.1%) |
| Opportunities and resources for research collaborations | 95 (17.4%) | 60 (15.6%) | 155 (16.7%) |
| Mentorship | 79 (14.5%) | 48 (12.5%) | 127 (13.7%) |
| Resources for internship possibilities | 73 (13.4%) | 46 (12%) | 119 (12.8%) |
| Other (included events and research experiences) | 2 (0.4%) | 0 (0%) | 2 (0.2%) |

**Abbreviations:** ISBT=International Society of Blood Transfusion; YP=young professionals; YPC=Young Professionals Council
